# A61 PRE-ALLOGENIC STEM CELL TRANSPLANT CLINICAL FACTORS AND THEIR ASSOCIATION WITH CHRONIC GRAFT VERSUS HOST DISEASE - A RISK FACTOR ANALYSIS FOR CHRONIC GVHD PREDICTION

**DOI:** 10.1093/jcag/gwab049.060

**Published:** 2022-02-21

**Authors:** A Alajmi, A Al Fedaghi, O Olaiya

**Affiliations:** McMaster University, Hamilton, ON, Canada

## Abstract

**Background:**

Allogeneic stem cell transplant is the only curative treatment for some hematological malignancies but is associated with significant toxicities and morbidities. The main long-term complication that is associated with decreased quality of life is chronic graft versus host disease (cGVHD) which can affect any organ system in the body. When clinicians are faced with the decision of transplanting patients many considerations must be taken into place. Unfortunately, despite counseling patients regarding possible toxicities and morbidities resulting from the allogeneic stem cell transplant including GVHD, we are unable to give accurate risks of cGVHD, especially organ-specific cGVHD, which might otherwise deter patients and clinicians from undertaking the transplant depending on their priorities that determine their quality of life.

**Aims:**

We sought to ascertain which prognostic factors could predict gastrointestinal (GI)/liver specific cGVHD.

**Methods:**

We conducted a retrospective case-control study including a consecutive sample of patients from a single specialty clinic from 2012–2017. Patients with biopsy proven GI/liver cGVHD based on pathology or determined by expert opinion reports were classified as cases and patients who underwent allogenic stem cell transplant without GI symptoms or liver enzyme elevations were controls. Data extracted included: age, gender, chemotherapeutics, donor status (related or unrelated), recipient and donor blood type, stem cell dose (x10^6^ cells), complete blood count and differential, and electrolytes. Logistic regression was used to analyze clinical factors associated with GI/liver specific cGVHD.

**Results:**

Our chart review retrieved 66 patients without GI/liver specific cGVHD and 55 patients that experienced GI/ liver cGVHD. The mean [SD] age of the sample was 50 [14] years and most of the participants in the study were men (59%). The most common cancer was acute myeloid leukemia. Univariate analysis revealed that male gender and stem cell dose were associated with GI/liver cGVHD. After adjusting for potential confounders, history of any acute GVHD demonstrated a statistically significant association with GI/liver cGVHD (adjusted OR [aOR]=2.7; 95% confidence interval=1.0–1.3; p=0.049), while male gender (aOR=2.7; 95% confidence interval=1.2–6.2; p=0.02) and stem cell dose (aOR=1.2; 95% confidence interval=1.0–1.3; p=0.037) remained statistically significant. The combination of these three clinical factors demonstrated fair discrimination (area under the curve=0.69). Other clinical factors were not significantly associated with GI/liver cGVHD.

**Conclusions:**

Male gender, previous episode of acute GVHD, and stem cell dose were strongly associated with gastrointestinal (GI)/ liver cGVHD among patients who receive allogeneic stem cell transplants.

**Funding Agencies:**

None

